# Bite count rates in free-living individuals: new insights from a portable sensor

**DOI:** 10.1186/s40795-018-0227-x

**Published:** 2018-05-18

**Authors:** Jimmy Alex, Dusty Turner, Diana M. Thomas, Andrew McDougall, Mirna W. Halawani, Steven B. Heymsfield, Corby K. Martin, Jenna L. Scisco, James Salley, Eric Muth, Adam W. Hoover

**Affiliations:** 1 Health and Public Affairs, University Of Central Florida, 838 Contravest Lane Winter Springs, Orlando, FL 32708 USA; 2 Department of Mathematical Sciences, United States Military Academy, West Point, New York, NY 10996 USA; 3 Center for Quantitative Obesity Research, Montclair State University, 1 Normal Ave, Montclair, Trenton, NJ 07043 USA; 4 Pennington Biomedical Research Center, Louisiana State University System, 6400 Perkins Road, Baton Rouge, LA 70808 USA; 5 Psychology Department, 344 Webb Hall, Eastern Connecticut State University, 83 Windham Street, Willimantic, CT 06226 USA; 6 Psychology Department, Clemson University, College of Business and Behavioral Sciences, 410 Brackett Hall, Clemson, SC 29364-0915 USA; 7 Department Electrical & Computer Engineering Department, Clemson University, Clemson, SC 29634-0915 USA

**Keywords:** Bite count rate, Sensor technology, Body mass index, Energy intake

## Abstract

**Background:**

Conclusions regarding bite count rates and body mass index (BMI) in free-living populations have primarily relied on self-report. The objective of this exploratory study was to compare the relationship between BMI and bite counts measured by a portable sensor called the Bite Counter in free-living populations and participants eating in residence.

**Methods:**

Two previously conducted studies were analyzed for relationships between BMI and sensor evaluated bite count/min, and meal duration. Participants from the first study (*N* = 77) wore the bite counter in a free-living environment for a continuous period of 14 days. The second study (*N* = 214) collected bite count/min, meal duration, and total energy intake in participants who consumed one meal in a cafeteria. Linear regression was applied to examine relationships between BMI and bite count/min.

**Results:**

There was no significant correlation in the free-living participants average bite counts per second and BMI (R^2^ = 0.03, *p* = 0.14) and a significant negative correlation in the cafeteria participants (*R*^*2*^ = 0.04, *p* = 0.03) with higher bite count rates observed in lean versus obese participants. There was a significant correlation between average meal duration and BMI in the free-living participants (*R*^*2*^ = 0.08, *p* = 0.01). Total energy intake in the cafeteria participants was also significantly correlated to meal duration (*R*^*2*^ = 0.31, *p* < 0.001).

**Conclusions:**

With additional novel applications of the Bite Counter, insights into free-living eating behavior may provide avenues for future interventions that are sustainable for long term application.

## Background

### What is already known about this subject?

Observational data on eating rates measured by self-report or through laboratory methods suggest that there is a relationship between higher eating rates and body mass index. There is some evidence that longer eating duration is associated with higher body mass index.

### What this study adds

- The analysis of data from wrist worn sensor that evaluates wrist motion did not reveal a relationship between bite count/min and body mass index.
- Data from the sensor indicates that there is a relationship between longer eating duration and body mass index.
- There is higher variance in bite count/min measured in a free-living environment compared to data collected in the cafeteria setting suggesting that extrapolation of eating behavior derived from laboratory settings to a free-living environment be viewed with caution.

## Introduction

Obesity is the result of energy intake exceeding energy expenditures. Controlling energy intake often involves reviewing daily and meal specific eating patterns. Meal-specific eating patterns have been analyzed using some measure of rate of food consumed. These measurements typically involve speed of bite counts taken (bite count/min) or mass consumed (g/min).

Feedback data on body weight and total daily intake has been used to successfully modify weight behavior [1]. However, these apps do not including timing and speed of eating behavior. Recently developed portable sensors that detect and provide real time ecologically valid data on eating behavior provide for the first time insight in free-living settings and detect whether results previously obtained in the laboratory or cafeteria setting translate to a free-living environment [2]. To date, eating rate measurements have required in clinic supervised eating, which limits participant sample size and observation of behaviors in a free-living environment. On the other hand, free-living measures of eating rates have long relied on self-report, which has not been demonstrated reliable [3].

Here we examine relationships between eating behavior and BMI from bite count/min obtained through a bite counter device worn on the wrist like a watch [4] that tracks a pattern of wrist roll motion to detect that the wearer has taken a bite of food. The capacity for the bite counter device to capture actual bites/min was originally validated on a total of 1675 bites taken by 47 people eating a single meal of their choice, finding an accuracy of 86% and a positive predictive value (precision) of 81% (true positives/(true positives + false positives))[4].

Terminology for measurement of eating speeds has been interchanged and is not well defined. Frequently, studies in which rates of food consumption activities, such as chews, [5, 6] bite counts [7] or swallows, [8] or even spoonfuls,[9] are measured and called bite count rates. Bite count rates can be measured in numerous ways. Additionally, the measured quantities are expressed in differing units such as mass or energy per unit time consumed or number of bite counts/chews/swallows per unit time. For example, in 1980, Kissileff et al. designed the innovative universal eating monitor to measure grams of food consumed per minute during a meal [10]. Others have used laboratory video recordings to calculate speed of bite counts (bite count/min)[11]. For clarity, we define *bite count rates* to represent bite count rate data measured by the Bite Technologies sensor.

The bite provides a unique window into eating patterns in a free-living environment.

## Methods

### Participants

Participants were recruited from the population of Clemson University and surrounding areas for two different studies [2, 12]. The first study evaluated bite counter records in a free-living population against self-reported intake (FREE-LIVING) [2]. The second study compared bite counter records with actual energy intake recorded in a cafeteria setting (CAFETERIA) [12]. Both studies were approved by the Clemson University Institutional Review Board.

### Free-living study

Detailed information on the original study was previously published [2]. Seventy-seven participants (age = 32.5 ± 12.4 y; BMI = 26.7 ± 5.9 kg/m^2^; 39 females, 38 males) with no history of eating disorders were told that the purpose of the study was to investigate if a new device could estimate the amount of food eaten during meals. History of eating disorder was determined by asking each participant the question: “Do you have a history of eating disorders (e.g., anorexia, bulimia)?” The participants self-reported yes or no. Each participant wore the bite counter for 14 days to obtain bite data for a total of 2975 meals (an average of 2.76 meals per person per day).

Subjects were asked to wear the bite counter continuously unless they were engaging in activities that could damage it, such as taking a shower. The bite counter recorded meal duration in seconds and the bite counts per second per meal. Participant intake was not guided and they freely consumed meals and snacks as part of their daily routine. The wrist-worn bite counter was returned to the study site for download and recording of the data.

### Cafeteria study

Additional study details have been previously published [12]. Participants with self-reported eating disorders were excluded from the study. Participants ate in a cafeteria on campus at Clemson University seated at a single, four-person customized table. The table was equipped with scales underneath place settings for monitoring weight changes, wrist-worn sensors for detecting bites, and cameras mounted in the ceiling that recorded videos of each meal. The participants were made aware of each recording device. The participants were allowed to select from a wide variety of meals (approximately 380 foods and beverages) in a cafeteria setting, which they self-selected and consumed while wearing the bite counter. A few of these items were available for nearly every session; these included all beverages, ice cream, pepperoni pizza, cheese pizza, hamburgers/cheeseburgers, shoestring French fries, chicken sandwiches, sandwich-bar sandwiches, and salads. The rest of the items, with some recurring once or twice, largely varied day to day. The participants were instructed to eat as much as they liked. A record was kept of all of the food items available for each day and time of the study.

Participants ate in groups of up to four and were allowed to schedule sessions with friends if they wished. Of the original sample of 280 participants, 44 ate with someone they knew. Four participants could not always be recruited for a single session: 136 ate in groups of four, 93 in groups of three, 46 in pairs, and 5 participants ate alone. As there was only one instrumented table in the cafeteria, participants always ate with their assigned cohort.

Energy intake (kcal) in the CAFTERIA participants was determined using a validated visual method [13, 14]. Food items selected by each individual participant were first identified from video footage. The selected portion of each food item was defined as a percentage of the reference serving size of the food item. The percentage of the selected portion of each food item consumed by each participant was then visually estimated by three raters. Energy intake (kcal) for each food item was calculated by multiplying the calorie content of the selected food item by the percentage of the reference portion selected and the percentage of the selected portion consumed which accounted for plate waste.

Of the original sample of 280 participants, 66 were absent from the final analysis due to data recording errors and outlier analysis for caloric intake and bite count. A reference database of 214 participants (age = 30.0 ± 12.1 y; BMI = 25.4 ± 5.6 kg/m^2^; 114 female, 100 male) containing simultaneous measures of total bite counts, total energy consumed per meal, meal duration, age, gender, and body mass index (BMI) was analyzed for this study.

### Statistical methods

All statistical analysis was performed in the statistical software SPSS (IBM, Armonk, NY 2012). The BMI-bite count/min plots were developed in Microsoft Excel (Seattle, WA 2011).

In the FREE-LIVING study, the number of meals was substantial (2975 meals) and varied across participants. Secondary data analysis was performed. In order to collapse data while ensuring integrity of results, analysis was performed by aggregating bite count rate information for each participant as average bite counts (bite count/min) over 14 days. Linear regression was conducted to test whether bite count rate (bite count/min) increased as a function of body mass index (BMI) or body weight. Linear regression was also performed to identify relationships between body mass index (BMI) and eating duration for both FREE-LIVING and CAFETERIA in Microsoft Excel (Seattle, WA, 2011).

In the CAFETERIA data, average bite count rates per individual were computed by averaging total bite counts over meal duration (bite count/min). In order to determine whether longer meal durations were related to higher energy intake, linear regression analyses examing relationships between meal duration and energy intake were performed. This analysis was only conducted in the CAFETERIA data because only the CAFETERIA study had objectively measured energy intake.

## Results

### Bite count rates in Free-living versus cafeteria

The range of bite count rates in the average bite count rate per individual from the FREE-LIVING study were [0.03,0.42] bite count/min. The range of bite count rates in the CAFTERIA study were [0.02, 0.14] bite count/min.

### Relationship between bite count rates and BMI

**Fig. 1:**
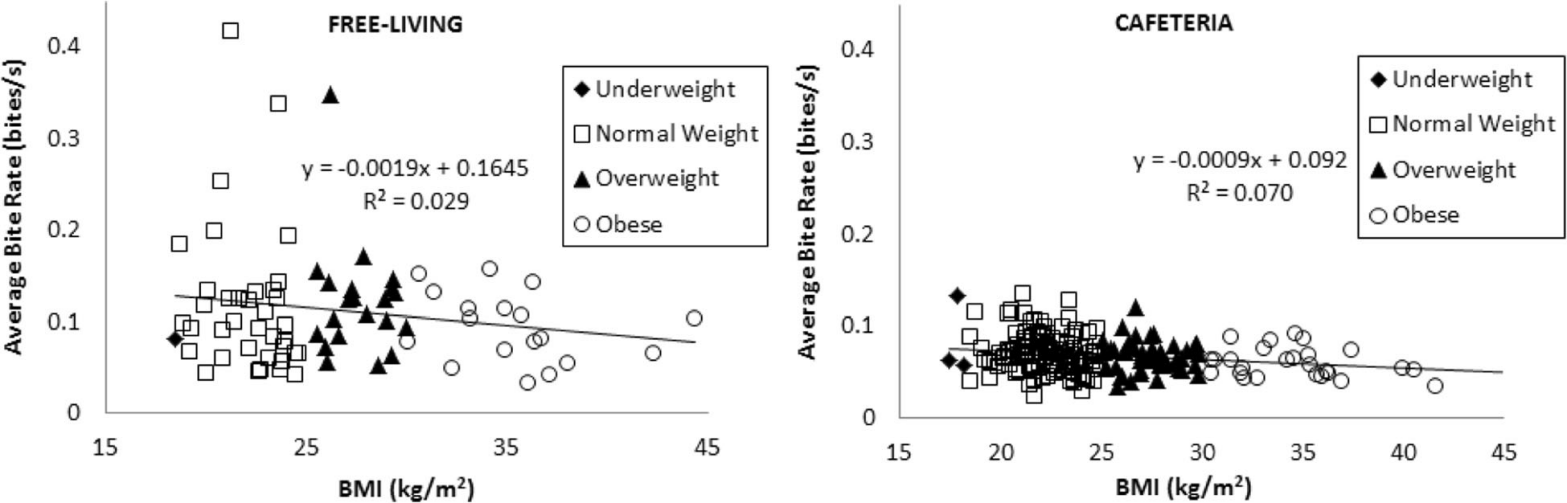
Average bite count/min in the FREE-LIVING (left panel) and CAFTERIA (right panel) studies against participant BMI. Average bite count/min in the FREE-LIVING subjects represents the average over all meals for individual participant over the 14-day period. There is no significant correlation between rate of bite counts and BMI in the FREE-LIVING participants and a weak negative correlation between rate of bite counts and BMI in the CAFETERIA participants

The correlation between average bite count rate and BMI (Fig. 1 Panel a and b) was small in both the FREE-LIVING and the CAFETERIA studies (FREE-LIVING R^2^ = 0.03, y = − 0.0019× + 0.1645, *p* = 0.64, CAFETERIA: R^2^ = 0.07, y = − 0.0009× + 0.0920, *p* = 0.03). The inverse correlation between BMI and bite count rate was significant in the CAFETERIA study. In both the CAFETERIA and FREE-LIVING studies, the fastest bite count rates were observed in lean participants.

### Relationship between meal duration and BMI

**Fig. 2:**
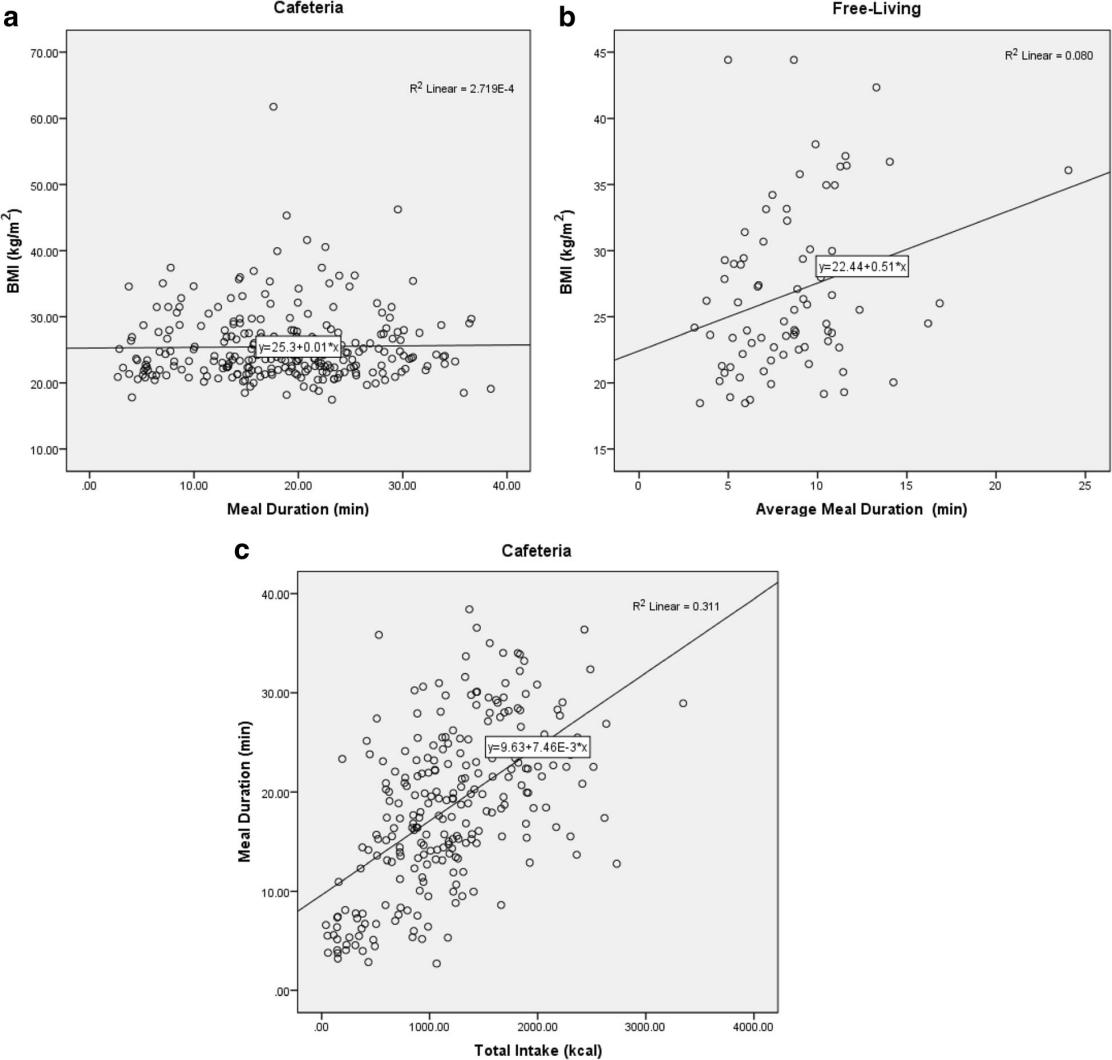
BMI versus average meal duration in the CAFETERIA (Panel **a**) and FREE-LIVING (Panel **b**) studies. A significant correlation (R^2^ = 0.331) between BMI and average meal duration is revealed in the FREE-LIVING subjects. Longer meal duration is correlated to higher energy intake in the CAFTERIA subjects (Panel **c**)

There was no significant correlation between meal duration and BMI in the CAFETERIA study (y = 0.01× + 25.3, R^2^ < 0.001, *p* = 0.22, Fig. 2 Panel a). The regression analysis in the FREE-LIVING data (y = 0.51× + 22.44, R^2^ = 0.08, *p* = 0.01) revealed a positive correlation between individuals who consumed food for longer durations and higher BMIs (Fig. 2 Panel b).

### Relationship between Total intake and meal duration

Eating for longer duration was positively correlated with higher total energy consumed in the CAFETERIA study (Fig. 2 Panel c). While meal duration was a statistically significant predictor of total energy consumed (*p* < 0.0001), the coefficient of determination was small (R^2^ = 0.11). The same analysis could not be conducted for the FREE-LIVING study since energy intake was not collected using objective methods.

## Discussion

Total energy intake is a function of bite count rate (bite count/min), mass of bite (kg/bite), and energy density of bite (kcal/bite) and duration of eating time. Manipulating these variables with the hope of changing total intake has been of great interest in the field; however, objectively measuring any of these variables in free-living subjects has been challenging. For the first time, the wearable Bite Counter allows us to observe and compare free-living bite count rate behavior with cafeteria results. In contrast to previous observational studies that rely on self-reported bite count rates,[15, 16] our results demonstrate that bite count rates are not correlated to BMI in free living subjects and only weakly correlated to BMI in cafeteria settings. Moreover, contrary to existing hypotheses, our analysis reveals a slight negative trend in BMI and bite count rates, that is, lean individuals have higher bite count rates than obese individuals. Because the bite counter also records eating time, the sensor revealed that free-living individuals with higher BMI were associated to longer eating duration. Additionally, we found a wider range of bite count rates in in the FREE-LIVING study compared to the CAFETERIA study. This may be due to participants eating in a contrived situation with groups of strangers in the CAFETERIA study. This difference is important because it demonstrates that a laboratory or cafeteria setting may not mimic the range of behavior in a free-living routine environment.

These results are concordant with conclusions derived from a chewing and swallowing sensor, which found that there appeared to be no correlation between mass per chew/swallow and BMI [1]. Although to our knowledge, experiments comparing time length of meals and BMI have not been conducted, several studies manipulated eating rates using universal eating monitors and compared meal duration in obese and lean participants [17, 18]. These studies found that the obese increase their eating time in response to directives to decelerate bites.

Our study has several limitations. The first limitation of the bite counter is that it cannot estimate bite size. Thus, individuals who eat longer may be ingesting smaller food mass per bite, which cannot be calculated by current methods. This ultimately limits the bite counter for deriving cumulative eating curves [19] which aggregate almost the entire set of eating behavior variables (speed, mass, and length) [20]. However, the bite counter applied with doubly labeled water to determine total energy intake per day offers an interesting avenue for triangulating intake data in free-living individuals and providing insights into eating behavior not previously accessible. Also, the bite counter must be turned on and off when the meal begins and ends. This presents a unique challenge and we note that at best the bite-counter represents a lower bound of eating duration. Nonetheless, it does provide objective evidence which perhaps could be combined with self-report and model tracking like those used in smart phone applications [21].

Another limiting factor is the fact that participants knew that they were being observed and the fact that many were eating with strangers could have altered total caloric intake and various other meal-dynamic variables (e.g. eating rate, bite count, bite size). Additionally, conversations were neither quantified nor qualified, and it is possible that conversational behavior may have been affected by the artificial environment. The results of our study suggest applying caution from extrapolating laboratory/cafeteria based conclusions to free-living eating behavior.

## Conclusion

In the context of this study, it does not appear that obese subjects on average have faster bite count rates than normal weight individuals. In fact, in both samples bite count rate was inversely associated with BMI. Individuals with a higher BMI ate longer than their lean counterparts. With additional novel applications of the bite counter, insights into free-living eating behavior may provide avenues for future interventions that are sustainable for long term application.

## Abbreviations

BMI: Body mass index
